# Supplementary figures and images for: HIF1α-PHD1-FOXA1 Axis Orchestrates Hypoxic Reprogramming and Androgen Signaling Suppression in Prostate Cancer
Source: Cells. 2025 Jul 2;14(13):1008. doi: 10.3390/cells14131008 (PMC12248476; doi:10.3390/cells14131008)

Liang et al. Figure S1

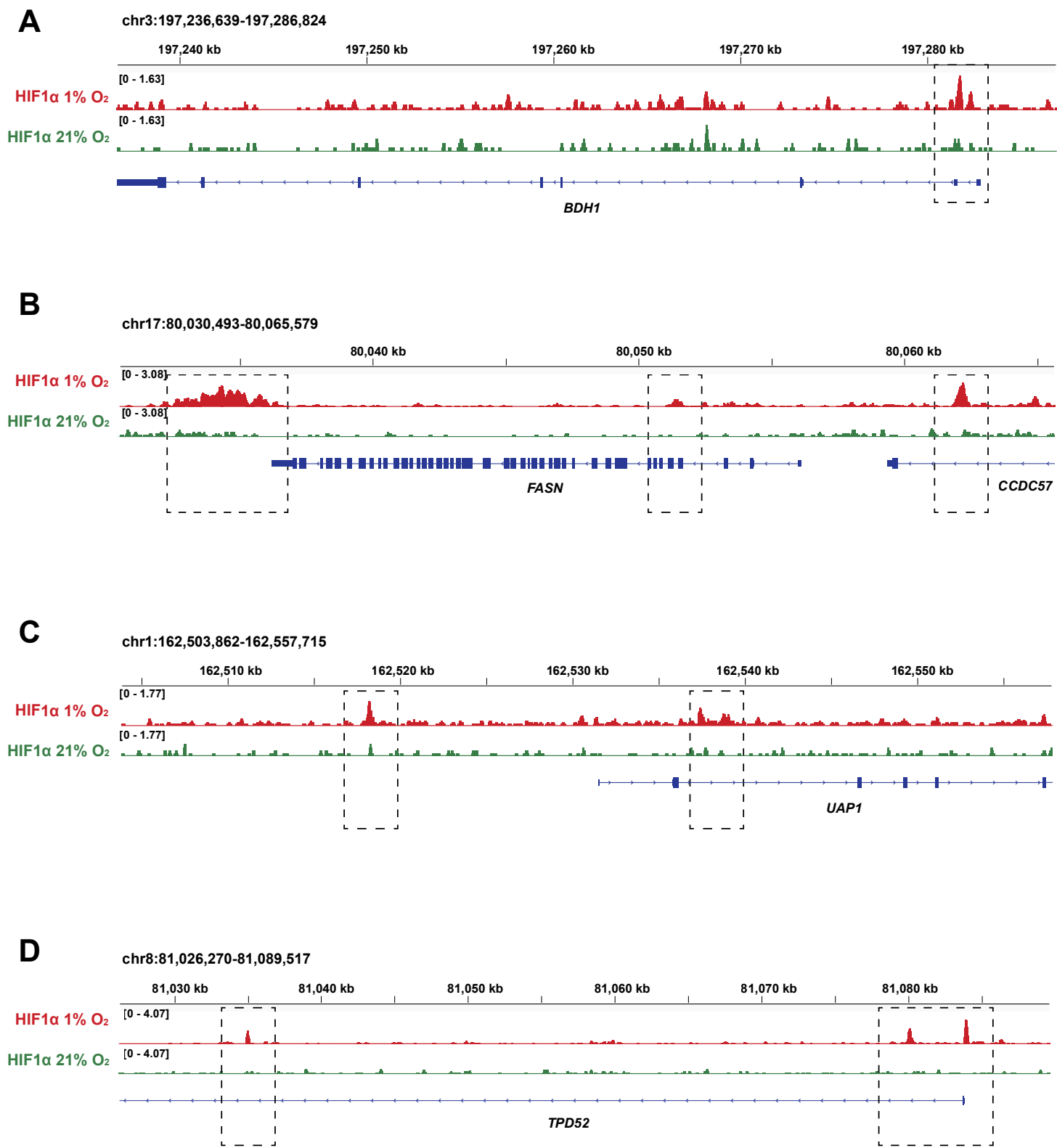

Liang et al. Figure S2

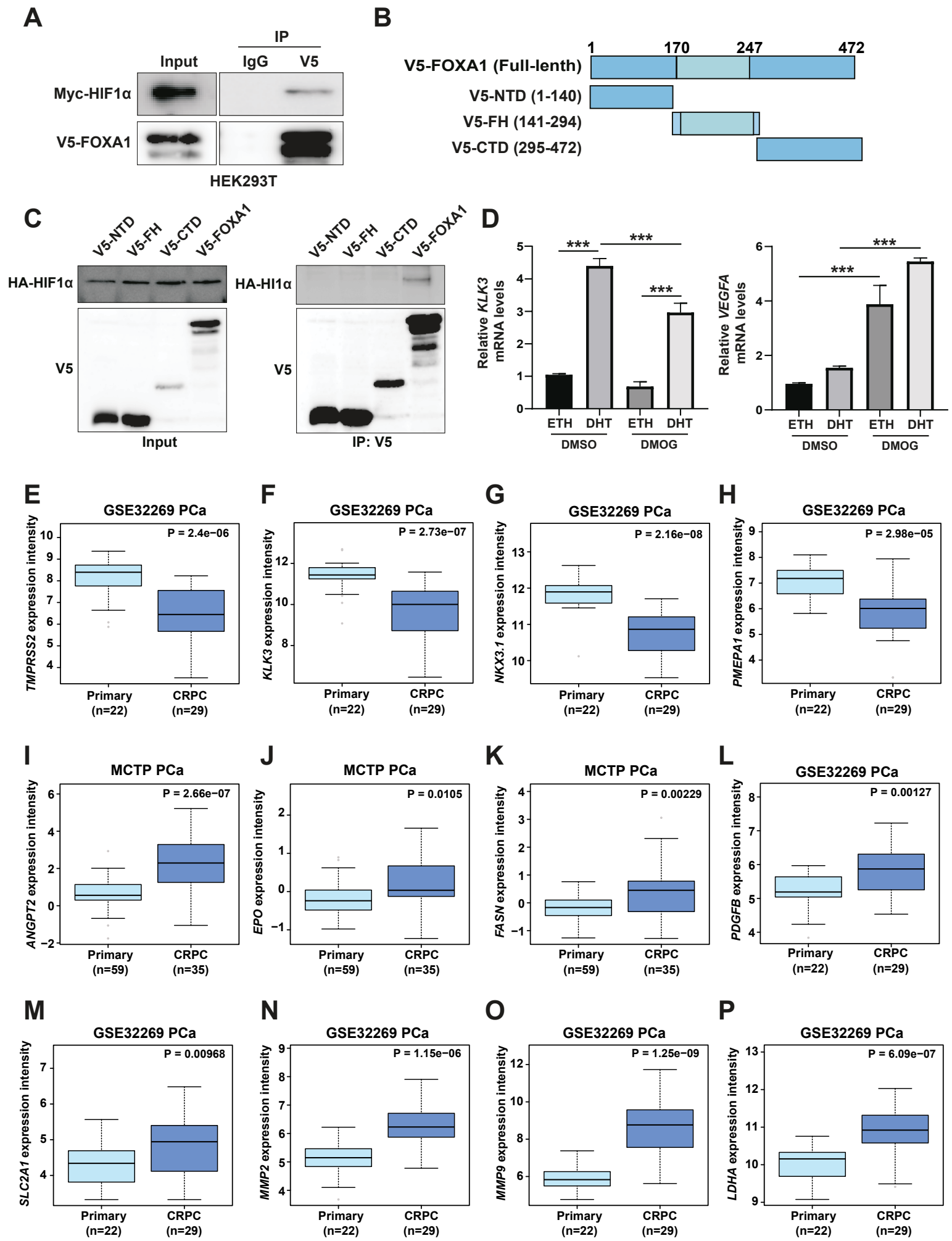

# Liang et al. Figure S3

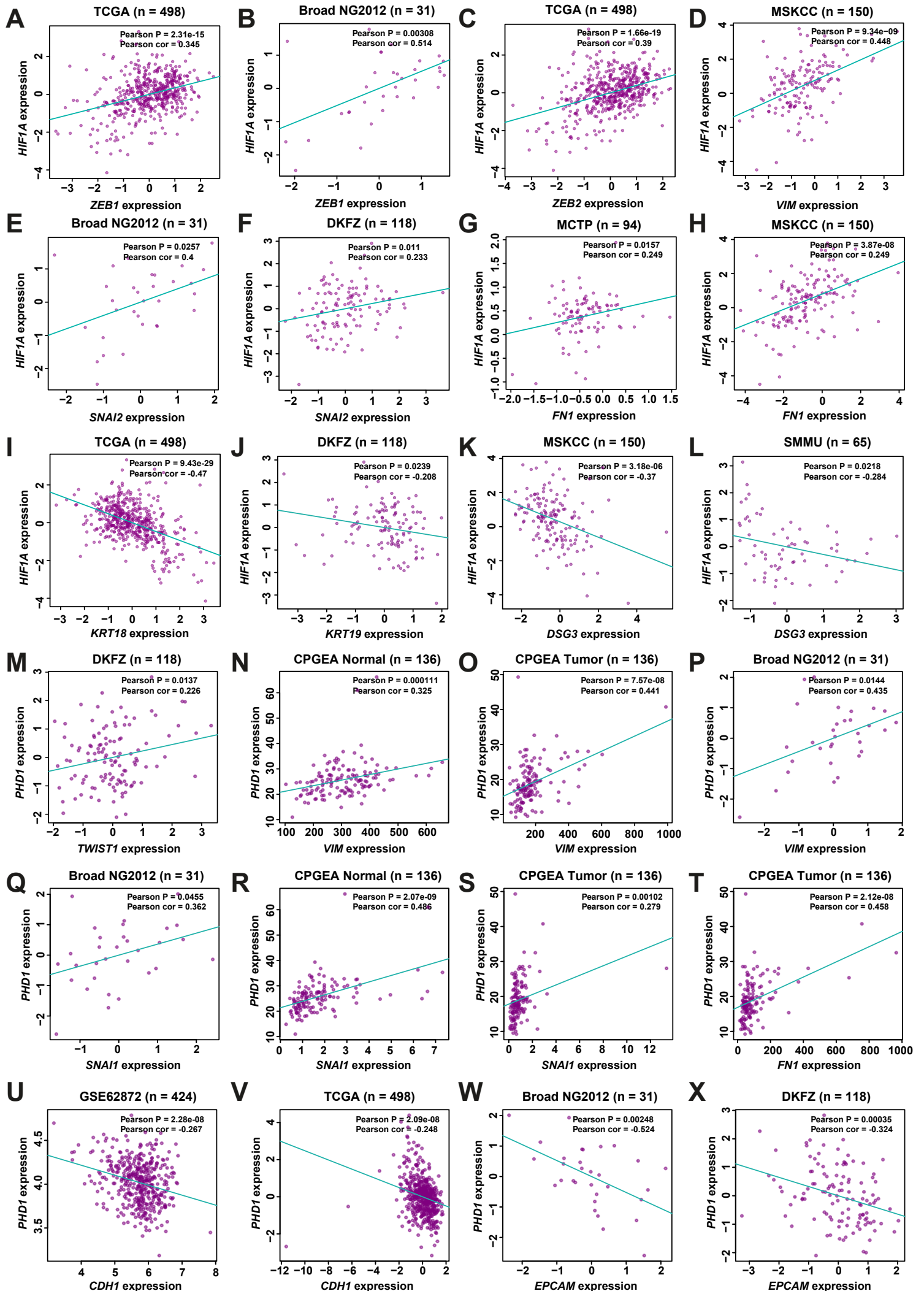

Supplement: Supplementary file 1 [file cells-14-01008-s001.zip › cells-3657260-supplementary.pdf]
